# Carnosic Acid Modulates Increased Hepatic Lipogenesis and Adipocytes Differentiation in Ovariectomized Mice Fed Normal or High-Fat Diets

**DOI:** 10.3390/nu10121984

**Published:** 2018-12-15

**Authors:** Yoon-Hee Lee, Whasun Lim, Mi-Kyung Sung

**Affiliations:** 1 Department of Food and Nutrition, College of Human Ecology, Sookmyung Women’s University, Chungpa-ro 47-gil 100, Yongsan-gu, Seoul 140-742, Korea; eiyoon88@naver.com; 2 Department of Biomedical Science, Catholic Kwandong University, 24 Beomil-ro 579 beon-gil, Gangneung-si, Gangwon-do 210-701, Korea; wslim@cku.ac.kr

**Keywords:** menopause, obesity, postmenopausal obesity, carnosic acid

## Abstract

As postmenopausal women experience a rapid increase in cardiovascular disease (CVD) risk with an increase in abdominal fat, dietary interventions to reduce CVD risk have been emphasized. This study was aimed at investigating the effect of a high-fat diet (HFD) in combination with an ovariectomy on liver and adipose tissue fat metabolism. The efficacy of carnosic acid (CA) supplementation in the suppression of HFD- and ovariectomy-induced obesity was also evaluated. Ovariectomized (OVX) or sham-operated mice at eight weeks of age were fed with a normal diet (ND), HFD, ND and 0.02% CA, or HFD and 0.02% CA for 12 weeks. All of the animals were sacrificed at the age of 20 weeks. The blood and tissue markers of the lipogenesis and adipocyte differentiation were measured. As expected, ovariectomy decreased the uterus weight and serum 17β-estradiol concentration. The HFD and ovariectomy significantly contributed to increases in the body weight and total fat mass, which were effectively inhibited by CA supplementation. The circulating concentrations of insulin, leptin, and TG (triglyceride) were significantly higher in the HFD group, and the concentrations were two to five times higher in the OVX and HFD group compared with those of the ND group. The CA supplementation significantly lowered the insulin, leptin, and TG concentrations in the OVX and HFD mice. The hepatic protein expressions of pAMPK and pACC were up-regulated by CA supplementation in OVX mice fed either ND or HFD. The expressions of hepatic SREBP1c and FAS mRNA were the highest in the OVX and HFD group, which were suppressed by CA supplementation. The adipose tissue PPARγ, aP2, and lipoprotein lipase (LPL) mRNA expressions were up-regulated by a HFD or ovariectomy, while they were significantly reduced in the mice fed a CA supplemented diet. The TNF-α and IL-6 mRNA levels in the adipose tissue were decreased by providing CA in the OVX groups. These results suggest that HFD and ovariectomy independently contribute to body fat accumulation, and CA effectively alleviated the ovariectomy-induced increases in lipogenesis and adipocyte differentiation. Further human trials are required in order to evaluate the efficacy of rosemary-derive CA as natural anti-adipogenic compounds, especially in postmenopausal women.

## 1. Introduction

Postmenopausal women are at a higher risk of developing metabolic abnormalities, possibly because of changes in hormonal milieu [1]. Estrogen regulates metabolic homeostasis and lipid metabolism, and studies have shown that the loss of the ovarian function accelerates adipose tissue accumulation, which is associated with insulin resistance and dyslipidemia [2,3,4,5]. Estrogen suppresses fat accumulation by modulating the key lipogenic genes and triglyceride (TG) synthesis both in the white adipose tissue (WAT) and liver [6,7]. While cardiovascular heart disease has been conceptualized as a male disease, a larger proportion of women live with CHD (cardiovascular heart disease) than men in aging populations, as a result of estrogen depletion [8]. Unfortunately, however, preventive strategies, including diet and physical activity, often do not prioritize gender differences. 

Diets that are high in fat elevate the risk of CHD through excess energy intakes, which increase the adiposity and adiposity-associated chronic inflammation. Postmenopausal obesity, not premenopausal obesity, has been also suggested as an important risk factor of breast cancer, indicating that postmenopausal women need more attention in order to reduce the risk of several life-threatening diseases associated with excess fat accumulation [9]. 

Hormone replacement therapy (HRT) has been suggested as an effective means to prevent menopause-induced weight gain [6]. In an earlier experimental study, chronic 17β-estradiol administration exerted a protective effect against body weight gain, glucose intolerance, and insulin resistance in mice that were fed a high-fat diet [10]. However, the benefits and harms of using HRT in postmenopausal women has remained controversial [11]. Based on more recent intervention studies using various types of estrogens in different subsets of postmenopausal women, the use of HRT was recommended in young postmenopausal women with no CHD risk factors [12]. Non-hormonal therapies to relieve menopausal symptoms have also been recommended, which might be important for those who do not benefit or face any possible harms from HRT.

Many plant polyphenols are well-known to possess anti-oxidant, anti-atherogenic, anti-cancer, and anti-inflammatory properties. Rosemary leaf extract was shown to limit weight gain and suppress the development of liver steatosis in mice fed a high-fat diet [13]. Carnosic acid (CA) is a primary phenolic compound found in the extracts of rosemary (Rosmarinus officinalis) leaves. It has been reported that CA reduces the body weight and the adipose tissue accumulation in overweight and obese mice [14,15,16]. However, the precise biological mechanisms associated with anti-adipogenic activity of CA have not been fully elucidated. 

In this study, we used ovariectomized animals deprived of estrogen, mimicking the postmenopausal status. The effects of the additional dietary fat intake and anti-adipogenic CA supplementation in ovariectomized (OVX) animals were evaluated in order to suggest a dietary regimen improving estrogen-associated metabolic alterations. 

## 2. Materials and Methods 

### 2.1. Animals and Experimental Design

Seven-week old female C57BL/6 mice from SLC Japan (Tokyo, Japan) were housed in a polycarbonate cage (three or four mice/cage), under controlled conditions of humidity (60 ± 5%), room temperature (23 ± 2 °C), and light (12 h light/dark cycle). The composition of the experimental diet was based on the American Institute of Nutrition (AIN)-93G. The fat sources of the normal diet (ND; 15% of fat calories) and high-fat diet (HFD; 45% of fat calories) were corn oil and lard. The CA group was fed a diet containing 0.02% CA (GBLS Cooperation Ltd, Seoul, Korea). Fresh food was provided every two–three days, and the food intake was monitored throughout the experiment. The body weight was monitored once a week. 

The animals were randomly divided into different experimental groups, as follows: Group 1 was sham-operated (SHAM) and fed a normal diet (ND; 15% of fat calories) (SHAM); Group 2 was ovariectomized (OVX) and fed ND (OVX and ND); Group 3 was OVX and fed ND containing 0.02% CA (OVX and ND and CA); Group 4 was SHAM fed a high fat diet (HFD; 45% of fat calories) (SHAM and HFD); Group 5 was OVX and fed a HFD (OVX and HFD); Group 6 was OVX and fed a HFD containing 0.02% CA (OVX and HFD and CA). The animals were maintained for 12 weeks. The mice were sacrificed at 20 weeks of age. All of the procedures were approved by the Institutional Animal Care and Use Committee of Sookmyung Women’s University (SM-IACUC-2013-0917-031).

### 2.2. Preparation of Blood and Tissue Samples

At the end of the experimental period, the animals were sacrificed. Serum was separated by centrifuging whole blood at 1550× *g* for 20 min, and was stored at −80 °C until assayed. The liver, spleen, uterus, and white adipose tissue (WAT), including the abdominal, gonadal, perirenal, and mesenteric WAT were removed and rinsed in normal saline. All of the samples were stored at −80 °C until the assays were performed. The tissue sections to be used for histological evaluation were stored in a 10% buffered neutral formalin.

### 2.3. Ovariectomy Procedure

The mice were anaesthetized with a mixture of zoletil and xylazine (47.5 mg/kg body weight, ip (intraperitoneal)). After 5 min, the anesthetized mice were placed on a hot water bag in order to prevent their body temperature from dropping during the operation. A 1.5 cm dorsal midline skin incision was made caudal to the posterior border of the ribs. The fascia was cleared away using blunt dissection, and the underlying muscle wall was pierced on both the right and left sides, 1 cm lateral to the spine. The ovary is located in a fat pad just beneath the muscles. Using forceps, the periovarian fat was gently grasped to lift and exteriorize the ovary. The uterine horn was returned into the abdomen, and the process was repeated on the other side. The skin incision was closed using wound clips. The sham-operated mice that were used as the control groups underwent the same procedures as the animals having with their ovaries removed, but their ovaries were not removed. 

### 2.4. Histological Tissue Analysis

Following the fixation of WAT with a 10% buffered formalin solution, the tissues were dehydrated in ethanol and then embedded in paraffin wax, sectioned, and stained with hematoxylin and eosin (H & E) for histologic analysis. 

### 2.5. Serum Measurements

The serum 17-β estradiol concentration was determined using a commercially available enzyme-linked immunosorbent assay (ELISA) kit (Calbiotech, Spring Valley, CA, USA), according to the manufacturer’s instruction. Serum leptin was measured by ELISA (R&D, Minneapolis, MN, USA). The serum insulin concentration was measured using an ELISA kit, according to the manufacturer’s protocol (Millipore Corp, Billerica, MA, USA). The serum free fatty acid (FFA) and triglyceride (TG) concentrations were determined using commercially available kits (Wako, OSAKA, Japan). 

### 2.6. Real-Time Quantitative PCR Analysis

Total RNA from the mouse liver and adipose tissue were extracted using a Trizol reagent (Invitrogen, Carlsbad, CA, USA), according to the manufacturer’s instruction. The adipose tissue was homogenized in 1ml of Trizol. The DEPC treated eppendorf tubes were shaken with 200 µL of chloroform for several seconds, and centrifuged at 18,020× *g*, 4 °C for 15 min. The RNA was precipitated with equal volumes of isopropanol and was pelleted by centrifugation. The total RNA was washed once with 70% ethanol, vaccum dried, and dissolved in DEPC-water. The total RNA (1 µg) was reverse-transcribed using a cDNA synthesis kit (PhileKorea Technology, Seoul, Korea) according to the manufacturer’s instruction. The real-time quantitative polymerase chain reaction (PCR) was performed on a 7500 Fast Real Time PCR system (Applied Biosystems, Foster city, CA, USA) using QuantiMix SYBR Kit (PhileKorea Technology, Seoul, Korea). Primers were designed using a nucleotide sequence and were synthesized by Bioneer (Bioneer, Daejeon, Korea). The following primers were used: SREBP1c, forward 5′-ATCTCCTAGAGCGAGCGTTG-3′ and reverse 5′-TATTTAGCAACTGCAGATATCCAAG-3′; FAS, forward 5′-CCCTTGATGAAGAGGGATCA-3′ and reverse 5′-GAACAAGGCGTTAGGGTTGA-3′; PPARα, forward 5′-CAGTGGGGAGAGAGGACAGA-3′ and reverse 5′-AGTTCGGGAACAAGACGTTG-3′; CPT-1, forward 5′-CCAGGCTACAGTGGGACATT-3′ and reverse 5′-GAACTTGCCCATGTCCTTGT-3′; PPARγ, forward 5′-GATGGAAGACCACTCGCATT-3′ and reverse 5′-AACCATTGGGTCAGCTCTTG-3′; aP2, forward 5′-GGGGCCAGGCTTCTATTCC-3′ and reverse 5′-GGAGCTGGGTTAGGTATGGG-3′; LPL, forward 5′-CTGCTGGCGTAGCAGGAAGT-3′ and reverse 5′-GCTGGAAAGTGCCTCCATTG-3′; TNF-α, forward 5′-ATGGGCTTTCCGAATTCAC-3′ and reverse 5′-GAGGCAACCTGACCACTCTC-3′; and IL-6, forward 5′-ACCAGAGGAAATTTTCAATAGG-3′ and reverse 5′-TGATGCACTTGCAGAAAACA-3′. The cycling conditions were as follows: 15 min at 95 °C, 40 cycles of 15 s each at 94 °C, and 30 s at 72 °C. The relative quantification was calculated using the Delta–Delta method [17]. The expression of the β-actin housekeeping gene was used to normalize the PCR reactions.

### 2.7. Protein Extraction and Western Blot Analysis

Tissue samples were homogenized for 20 min on ice in a PRO-PREP™ protein extraction solution (Intron Biotechnology, Gyeonggi, Korea), and were centrifuged (16,600× *g*, 10 min, 4 °C). The protein content was determined against a standardized control, using a Bio-Rad Protein Assay kit (Bio-Rad Laboratories Inc., Hercules, CA, USA). A total of 50 µg of protein from each sample was separated by 4–12% and 6% sodium dodecyl sulfate polyacrylamide gel electrophoresis, and was transferred to PVDF membranes (GE Healthcare, Mickleton, NJ, USA). The membranes were blocked with 2% skim milk (Bioworld, Dublin, OH, USA) and incubated with specific antibodies for pAMPK (dilution 1:1000; Cell signaling, Boston, MA, USA), AMPK (dilution 1:1000; Cell signaling, Boston, MA, USA), pACC (dilution 1:2000; Cell signaling, Boston, MA, USA), ACC (dilution 1:2000; Cell signaling, Boston, MA, USA), and β-actin (dilution 1:4000; Sigma, St. Louis, MO, USA). Anti-rabbit IgG or anti-mouse IgG conjugated with peroxidase (Sigma, St. Louis, MO, USA) was used as the second antibody. The membranes were washed with PBS/Tween 20 (PBST) containing 0.1% Tween20 (Sigma, St. Louis, MO, USA). The reactive bands were visualized with an enhanced chemiluminescence (ECL) system (GE Healthcare, Mickleton, NJ, USA). Stripping was checked by re-exposure to the enhanced ECL and was detected by LAS 3000 (Fujifilm, Tokyo, Japan). The membranes were subsequently blocked and reproved. The intensity of the bands was quantified using a Bio-Rad GS-800 densitometer equipped with the Quantity One program (Bio-Rad Laboratories Inc., Hercules, CA, USA).

### 2.8. Statistical Analysis

The statistical analysis was performed using the SAS package (release 9.3, SAS Institute Inc., Cary, NC, USA). The data are expressed as the means ± standard deviation (SD). The one-way analysis of variance (ANOVA) and the Duncan’ multiple test were used to determine the statistical differences between the treatment groups. The Student’s *t*-test was used to analyze the differences between the two groups. A value of *p* < 0.05 was considered statistically significant.

## 3. Results

### 3.1. Body and Fat Tissue Weights

To confirm that OVX was successfully carried out, the uterus weight and serum concentration of estradiol were measured (Table 1 and Table 2). The OVX groups exhibited a significantly lower uterus weight and circulating concentrations of estradiol compared to the SHAM groups. HFD or ovariectomy accelerated body weight gain (Table 1). The animals in the OVX and HFD group weighed the most among all of the experimental groups. Both in the OVX and ND, and OVX and HFD groups, the CA supplementation significantly suppressed the weight gain of the animals. 

The body fat was divided into the mammary fat pad, retroperitoneal fat, gonadal fat, perirenal fat, and mesenteric fat, according to their locations, and their respective weights were measured. Th results indicated that both HFD and ovariectomy significantly increased the respective fat tissue weight. CA supplementation suppressed the total and specific fat tissue weights, and a statistically significant efficacy was observed in the OVX mice fed with HFD. 

### 3.2. Serum Measurements

Table 2 shows the effects of ovariectomy, HFD, and CA supplementation on the concentrations of serum leptin, insulin, triglycerides (TG), and free fatty acids (FFA). All of the four adiposity-associated biomarkers were affected by OVX, HFD, and CA supplementation. However, HFD showed a higher extent of increment in the insulin and TG concentrations compared to ovariectomy. Concomitantly, the OVX and HFD group had highest serum concentrations of insulin, leptin, TG, and FFA. The CA supplementation significantly reduced the circulating concentrations of leptin, TG, and FFA in the ND group animals. The OVX and HFD and CA group showed significantly lower concentrations of insulin, leptin, TG, and FFA compared with OVX and HFD group.

### 3.3. Expression of Liver AMPK and ACC Protein

**Figure 1:**
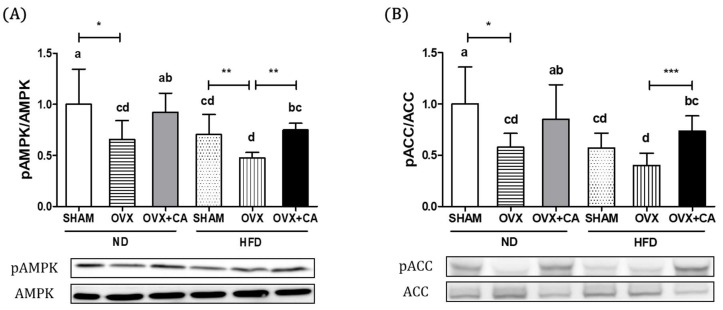
Effects of carnosic acid (CA) on the expression of phosphorylated AMPK and ACC in liver tissue. The expression of phosphorylated AMPK (**A**) and ACC (**B**) were measured by Western blot analysis. Values are presented as the mean ± standard deviation (SD) of the ratio between pAMPK and AMPK. Bars with letters (a, b, c, and d) are significantly different from each other, as determined by Duncan’s multiple range test (*p* < 0.05, * *p* < 0.05, ** *p* < 0.01, *** *p* < 0.001). SHAM—sham-operated; OVX—ovariectomized; ND—normal diet; HFD—high fat diet; CA—carnosic acid.

To elucidate molecular mechanisms, liver tissue expressions of AMPK, p-AMPK, ACC and p-ACC proteins were determined. The phosphorylation of AMPK promotes the β-oxidation of fatty acids and inhibits lipogenesis by up-regulating PPARα and CPT-1 expression and down-regulating SREBP1c and FAS expression. As expected, the phosphorylation of AMPK was further decreased after provision of HFD (Figure 1). The AMPK phosphorylation was significantly lower in OVX + ND and OVX + HFD groups compared to that of the SHAM + ND control group. CA supplementation significantly increased the expression of pAMPK/AMPK both in ND group animals and HFD group animals. The activation of AMPK down-regulates the phosphorylation of ACC, a gene involved in fatty acid synthesis. CA supplementation significantly accelerated the phosphorylation of ACC significantly increasing the ratio of pACC to ACC in animals of OVX + ND and OVX + HFD groups. 

### 3.4. Expression of Liver Lipogenesis-Related Genes

**Figure 2:**
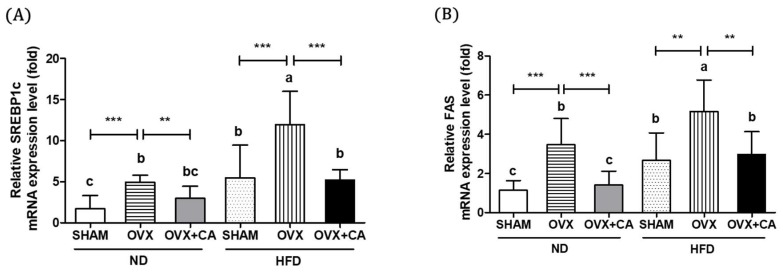

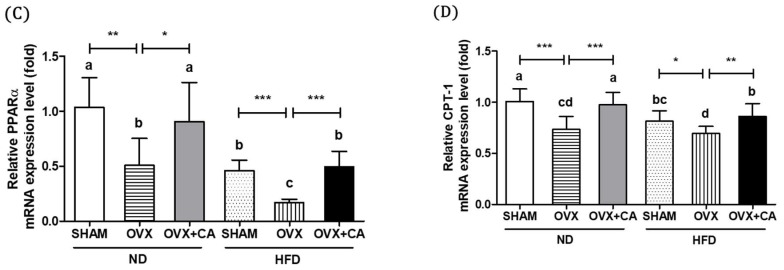
Effect of CA on the hepatic SREBP1c, FAS, PPARα, and CPT-1 mRNA expression. The expressions of the SREBP1c (**A**), FAS (**B**), PPARα, (**C**) and CPT-1 (**D**) mRNA were measured by real-time PCR analysis. The values are presented as the mean ± SD. Bars with letters (a, b, c, and d) are significantly different from each other, as determined by Duncan’s multiple range test. (*p* < 0.05, * *p* < 0.05, ** *p* < 0.01, and *** *p* < 0.001). SHAM—sham-operated; OVX—ovariectomized; ND—normal diet; HFD—high fat diet; CA—carnosic acid.

Figure 2A,B shows the expression levels of SREBP1c and FAS, which are associated with the lipogenic activity of the liver. The study results clearly show that both OVX and HFD independently increased the expression of these two lipogenic genes. The level of expression was highest in the OVX and HFD group. CA supplementation significantly reduced the expression level of SREBP1c mRNA in the animals from the OVX and HFD group, whereas the expression levels of FAS mRNA were reduced in the animals from the OVX and ND, and OVX and HFD groups. HFD further decreased the lowered expression levels of the β-oxidation genes, including PPARα and CPT-1 mRNA, in the OVX mice, as shown in Figure 2C,D. The CA supply induced significant increases in the expression of PPARα and CPT-1 both in the OVX and ND, and OVX and HFD groups. These results indicate that HFD and/or OVX induced hepatic lipogenesis, while CA suppressed fatty acid synthesis and stimulated β-oxidation. 

### 3.5. Expression of Adipocyte Differentiation- and Fat Accumulation-Related Genes in WAT

**Figure 3:**
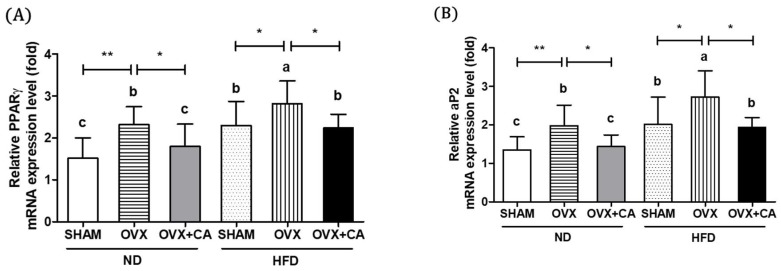

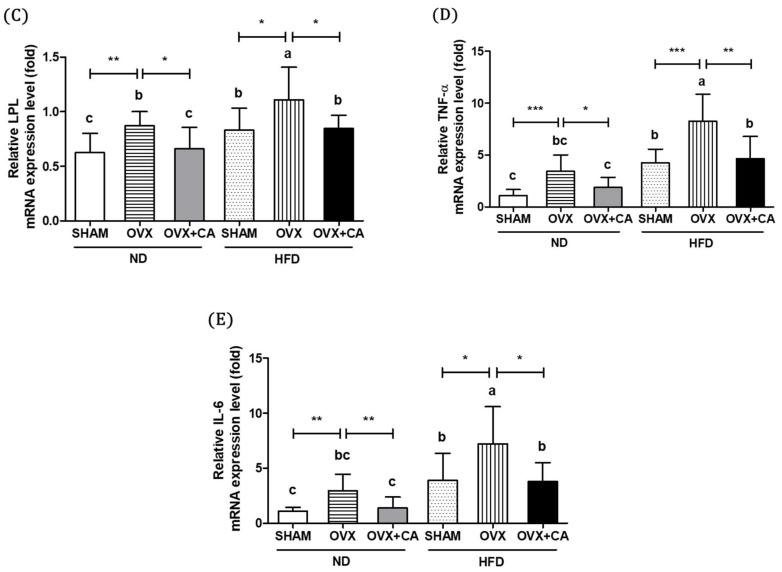
Effect of CA on the mRNA expression of adipocyte differentiation- and accumulation-related genes and markers inflammation. The expressions of the PPARγ (**A**), aP2 (**B**), LPL (**C**), TNF-α (**D**), and IL-6 (**E**) mRNA were measured using a real-time PCR analysis. Values are presented as the mean ± SD of the relative expression. Means with letters (a, b, c, and d) within the column are significantly different from each other, as determined by Duncan’s multiple range test (*p* < 0.05, * *p* < 0.05, ** *p* < 0.01, *** *p* < 0.001). SHAM—sham-operated; OVX—ovariectomized; ND—normal diet; HFD—high fat diet; CA—carnosic acid.

To investigate the effects of HFD, OVX, and CA on adipocyte differentiation and fat accumulation, we measured the expression levels of the key genes related to adiposity, including PPARγ, aP2, and lipoprotein lipase (LPL), in WAT. PPARγ is a transcription factor involved in adipocyte differentiation and regulates the expression of adipocyte markers such as aP2, a carrier protein for fatty acids. LPL regulates the peripheral TG uptake. The results showed that the HFD accelerated the OVX-induced increases in the PPARγ, aP2, and LPL mRNAs (Figure 3A–C). In both the OVX and ND, and OVX and HD groups, the CA supplementation down-regulated the expressions of PPARγ, aP2, and LPL to the control levels.

### 3.6. Expression of WAT Pro-Inflammatory Genes

The TNF-α and IL-6 expressions in WAT were significantly increased in the HFD and OVX groups (Figure 3D,E). As expected, the OVX and HFD group showed the highest expression levels compared to the other groups, and the CA supplementation significantly suppressed the OVX, or HFD induced the up-regulation of TNF-α and IL-6. 

### 3.7. Adipocyte Size: Histological Analysis

**Figure 4:**
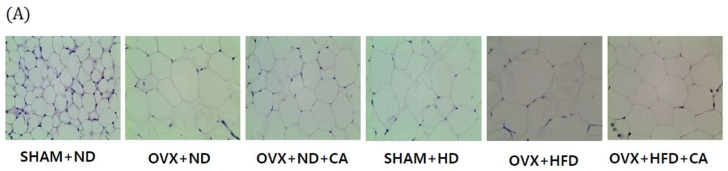

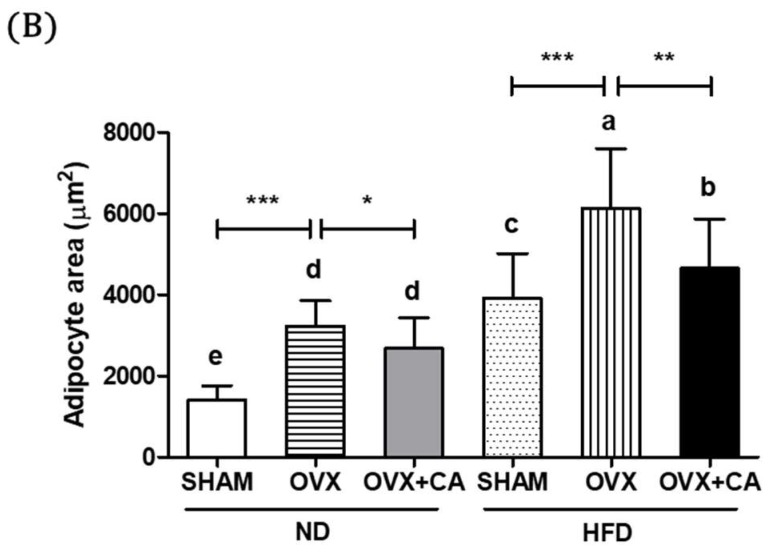
Effect of CA on adipocyte morphology (**A**) and size (**B**). The adipose tissue sections obtained at the end of the 20 weeks were stained with hematoxylin and eosin (×400). Values are presented as the mean ± SD. Bars with letters (a, b, c, and d) are significantly different from each other, as determined by Duncan’s multiple range test (*p* < 0.05, * *p* < 0.05, ** *p* < 0.01, *** *p* < 0.001). SHAM—sham-operated; OVX—ovariectomized; OVX + CA—ovariectomized + carnosic acid; ND—normal diet; HFD—high fat diet; CA—carnosic acid.

The HFD and OVX significantly increased the size of abdominal fat adipocytes (Figure 4). Although OVX and HFD independently affected the size of the adipocytes, a marked increase was observed in the OVX and HFD group. The CA-fed mice showed significantly reduced adipocyte sizes in the OVX and HFD group. 

## 4. Discussion

One of the major health issues in postmenopausal women is weight gain associated with an increase in body fat mass. The lack of estrogen has been suggested as a causative factor in postmenopausal body weight gain and predominant abdominal fat accumulation. Despite the fact that visceral obesity is a key risk factor for postmenopausal CVD and certain types of cancer, sex- and age-specific prevention strategies are undermanaged [5]. 

Experimental studies have indicated that estrogen depletion leads to metabolic disturbances, especially in the acceleration of fat synthesis. Many observational studies have found that postmenopausal women receiving estrogen therapy showed a lower risk of developing CVD. However, a large scale randomized controlled trial suggested that estrogen and progestin therapy did not alter the rate of CHD events in postmenopausal women with established coronary disease [18,19,20,21]. A subsequent randomized primary-prevention trial from the Women’s Health Initiative reported that the combined use of estrogen and progestin may increase the risk of CVD [22]. Controversial findings between clinical trials and observational studies have been explained by many possible confounders. Also, despite the fact that estrogen depletion is directly associated with dramatic weight gain in this population, few studies have been conducted to modify the metabolic disturbance in postmenopausal women through dietary modifications. A study conducted based on the WHI clinical trial showed that a low fat dietary pattern in accordance with increased fruits, vegetables, and grains did not significantly reduce the risk of CVD, stroke, or CHD in postmenopausal women, with modest effects on risk factors, suggesting the needs for further dietary or lifestyle modification intervention studies [23].

In this study, we examined the effects of a diet high in fat and/or estrogen depletion, because multiple estrogen and estrogen receptor-mediated signals on energy metabolism have been emphasized in order to explain visceral obesity in postmenopausal women. Also, the efficacy of the anti-adipogenic compound, CA, and its supplementation to suppress fat accumulation was determined. CA was shown to protect hepatic steatosis and its related metabolic disorders, including hyperglycemia, insulin resistance, and the blood lipid profile, in HFD-fed mice [16]. Our data demonstrated that both HFD and OVX develop significant metabolic changes, including an increase in body fat and increases in the circulating concentrations of insulin, leptin, lipids, and pro-inflammatory cytokines. These changes were associated with the expression of genes regulating lipogenesis. It was also demonstrated that the body weight and total fat mass were significantly higher in the OVX and HFD group compared with those of the other groups, indicating that an excess energy intake may impose a greater risk to developing obesity and related metabolic diseases under an estrogen-deficient state. Furthermore, we confirmed that CA contributes to the suppression of HFD- and OVX-induced metabolic changes in this experimental animal model. 

WAT is the major energy reservoir in mammals and plays an important role in the maintenance of whole-body energy homeostasis [24]. It has been reported that body weight and fat distribution differ by sex hormone concentrations. Women in the reproductive period of life have more subcutaneous body fat, which is deposited in the hips and thighs [25]. However, postmenopausal women deposit more visceral fat [26]. This study showed that visceral fat, which is composed of retroperitoneal, gonadal, perirenal, and mesenteric fat, was significantly increased in the OVX and HFD animals. In general, the increased visceral fat in the postmenopausal period strongly affect the high free fatty acid levels, and it led to insulin resistance and metabolic syndrome [27,28]. Moreover, the visceral fat is associated more with the increasing inflammatory responses, producing a higher amount of pro-inflammatory leptin, TNF-α, and IL-6 than subcutaneous fat [29,30]. In a previous study, estrogen replacement therapy suppressed abdominal fat accumulation, but not the subcutaneous fat mass [31].

The diet supplemented with 0.2% CA significantly reduced the body weight and the accumulation of subcutaneous mammary fat pads, as well as visceral fat, including retroperitoneal, gonadal, perirenal, and mesenteric fat, in OVX and OVX and HFD groups. However, the efficacy was statistically significant only in the OVX and HFD group. As postmenopausal women deposit more visceral fat causing metabolic complications, CA might be a useful compound to maintain metabolic homeostasis through less visceral fat accumulation. We have previously reported that CA suppresses hepatic fat accumulation by down-regulating the expression of lipolysis-related genes [32]. Also, CA treated 3T3-L1 adipocytes showed less triglyceride accumulation and glycerol 3-phosphate dehydrogenase activity [33]. However, the mechanistic explanation of CA to ameliorate obesity induced by HFD and/or OVX is still poorly elucidated.

To further investigate the mechanism underlying the adipogenic effects of HFD and OVX, and the anti-obesity effects of CA supplementation on accelerated fat accumulation by OVX or HFD, we measured the AMPK-induced signaling associated with fat synthesis and oxidation. AMPK, a serine threonine composed of α subunit and regulatory β and γ subunits, is a cellular energy sensor that regulates glucose and lipid metabolism [34]. The activation of AMPK regulates lipid metabolism via multiple downstream targets involved in fatty acid synthesis and β-oxidation [35]. The inactivation of AMPK has been shown to induce fat accumulation and decrease insulin sensitivity [36,37]. Previous studies observed the under-activation of AMPK during the development of obesity [38,39]. It is also reported that ovarian hormones may contribute to AMPK activation and the levels of AMPK phosphorylation are reduced by ovarectomy [40,41]. Based on these observations, recent studies have focused on the role of the AMPK and AMPK activators, to gain insight into the efficacy against obesity and obesity-related diseases. AMPK activation is induced by various agents including 5-aminoimidazole-4-carboxamide-1-β-D-ribofuranoside (AICAR), metformin, and thiazolidinediones (TZDs). Importantly, these numerous supplementations have detrimental effects, for example, AICAR inhibits mitochondrial oxidative phosphorylation independently of AMPK, and TZDs have a risk of cardiovascular diseases [42,43,44]. Therefore, it has been a challenge to develop an AMPK activator without side effects. The results from this study suggest that CA may act as an effective AMPK activator. Although CA has not been subjected to clinical intervention, a previous study reported that the oral lethal dose (LD50) of CA was 7100 mg/kg body weight for mice and CA at the level of 150 mg/day/kg body weight did not affect the clinical chemistry parameters, suggesting that CA has a relatively low oral toxicity profile [45]. We used the supplementation of 0.02% CA in the diet (oral intake of 10–20 mg/day/kg body weight), and no clinical signs of adverse events were observed. 

A previous study demonstrated that estrogen suppresses the expression of SREBP1c, which is a transcription factor and promotes the expression of FAS, which is a lipogenic gene, in the adipose tissue, muscle, and liver [6]. The increased amount of WAT in obesity may attribute to the increased circulating concentrations of TG, FFA, insulin, and leptin. Increased concentration of FFA induces insulin resistance and elevates the expression of the hepatic lipogenic genes, including SREBP1c and FAS in the liver [46]. On the other hand, it was demonstrated that PPARα and its target genes, including CPT-1, were significantly decreased in HFD-induced obesity [16]. PPARα, a transcription factor regulating lipid metabolism genes, is activated by AMPK phosphorylation. It has been reported that hepatic PPARα and CPT-1 up-regulate fatty acid β-oxidation, inhibiting fat accumulation [47]. In this study, CA suppressed the expression of hepatic lipogenesis genes, such as SREBP1c and FAS, which were highly expressed by HFD and OVX, and the up-regulated expression of β-oxidation-associated PPARα and CPT-1. These results suggest that CA has the beneficial effect of hepatic lipogenesis and β-oxidation via the AMPK mediated lipid metabolism pathway.

Adipogenesis is initiated by the production of PPARγ, which stimulates adipocyte differentiation and the apoptosis of large adipocytes via the activation of down-stream targets, including aP2 and LPL [48]. A previous study suggests that estrogen has anti-inflammatory and atheroprotective effects via regulating the PPARγ-mediated pathway [49]. In this study, the expression of adipocyte differentiation and fat accumulation related-genes, including PPARγ, aP2, and LPL, were significantly increased by HFD and/or OVX, and decreased by CA supplementation. In addition, the histological observations demonstrate that a diet supplemented with CA effectively decreased adipocyte morphology and size, which were increased by HFD and/or OVX. Although we have not measured the efficacy of CA to activate brown adipose tissue (BAT) metabolism, a recent study indicated that the decreased Nrf1-mediated proteasomal activity of BAT in Nfe2l1^ΔBAT^ mice exhibited the diminished cellular integrity of BAT, including the whitening of BAT [50]. Increased visceral fat is related with metabolic diseases and inflammation, in association with an increased concentration of pro-inflammatory leptin, TNF-α, and IL-6 by increased adipose tissue, rather than subcutaneous fat [29]. The expression of TNF-α mRNA in adipose tissue was significantly increased by HFD and/or OVX. Also, the IL-6 mRNA expression level was affected by HFD, however, a significant effect of OVX was shown only in the HFD-fed mice. Also, the abdominal fat TNF-α and IL-6 mRNA expressions were significantly increased in the OVX and HD group compared with those of the other groups. As expected, these inflammatory markers were significantly decreased by CA supplementation.

Apart from lipid synthesis and oxidation, previous studies also suggested that CA-rich rosemary extract and CA inhibited gastric and pancreatic lipase activity, respectively, indicating that a part of the anti-adipogenic activity of CA might be derived from a reduction in fat absorption [14,51].

These results suggest that rosemary-derived CA suppressed the HFD or OVX-induced increases in excess fat accumulation by regulating the important molecular mediators associated with fat synthesis and oxidation. CA also stimulated adipocyte differentiation. 

## 5. Conclusions

In conclusion, the present study clearly demonstrated that a high-fat diet and/or systemic loss of estrogen induce ectopic fat accumulation and adipocytes differentiation, suggesting that excess energy supply and estrogen deprivation exert a major influence on postmenopausal obesity and related metabolic disturbances. In the meantime, CA suppressed abnormal lipid metabolism, fat accumulation, and adiposity in OVX mice, suggesting the possible use of CA as a functional compound to alleviate metabolic disturbances in postmenopausal women by inhibiting excess visceral adipose tissue accumulation and adipocytes differentiation.

## Figures and Tables

**Table 1:** Body weight, uterus weight, and percent body fat of experimental animals.

	Group					
	SHAM and ND	OVX and ND	OVX and ND and CA	SHAM and HFD	OVX and HFD	OVX and HFD and CA
Body weight(g)	22.82 ± 1.84 ^c^	26.70 ± 1.36 ^b^	24.45 ± 1.12 ^c^	28.11 ± 3.45 ^b^	32.32 ± 4.79 ^a^	28.06 ± 3.15 ^b^
Uterus weight (g)	0.46 ± 0.10 ^a^	0.11 ± 0.10 ^c^	0.11 ± 0.12 ^c^	0.36 ± 0.09 ^b^	0.08 ± 0.09 ^c^	0.11 ± 0.14 ^c^
Total fat (% of BW ^1^)	3.18 ± 1.24 ^d^	7.02 ± 1.92 ^b,c^	5.25 ± 2.02 ^c,d^	8.61 ± 3.71 ^b^	13.27 ± 6.88 ^a^	9.49 ± 4.09 ^b^
Retroperitoneal fat	0.77 ± 0.44 ^d^	2.04 ± 0.59 ^b,c^	1.50 ± 0.76 ^c,d^	2.44 ± 1.13 ^b,c^	3.88 ± 2.08 ^a^	2.95 ± 1.52 ^a,b^
Gonadal fat	0.60 ± 0.37 ^d^	1.23 ± 0.56 ^b,c,d^	0.99 ± 0.50 ^c,d^	1.69 ± 0.66 ^b^	2.52 ± 1.49 ^a^	1.58 ± 0.76 ^b,c^
Perirenal fat	0.40 ± 0.12 ^d^	1.03 ± 0.33 ^c^	0.70 ± 0.27 ^c,d^	1.17 ± 0.48 ^c,b^	2.15 ± 1.10 ^a^	1.54 ± 0.72 ^b^
Mesenteric fat	0.57 ± 0.35 ^d^	1.08 ± 0.34 ^b,c^	0.78 ± 0.32 ^c,d^	1.34 ± 0.40 ^b^	1.78 ± 0.86 ^a^	1.15 ± 0.55 ^c,b^
Mammary fat pad	0.83 ± 0.21 ^d^	1.63 ± 0.49 ^b,c^	1.28 ± 0.42 ^c,d^	1.97 ± 0.68 ^b,c^	2.95 ± 1.75 ^a^	2.07 ± 0.79 ^b^

Values are presented as the mean ± standard deviation (SD). Means with letters (a, b, c, and d) within the column are significantly different from each other, as determined by Duncan’s multiple range test (*p* < 0.05). The body fat contents were calculated as percentages of the Bbody weight. SHAM—sham-operated; OVX—ovariectomized; ND—normal diet; HFD—high-fat diet; CA—carnosic acid, ^1^: Body weight.

**Table 2:** Serum concentrations of insulin, leptin, triglyceride, and free fatty acids.

Parameter	Group					
	SHAM and ND	OVX and ND	OVX and ND and CA	SHAM and HFD	OVX and HFD	OVX and HFD and CA
Estradiol (pg/mL)	18.92 ± 2.37 ^a^	5.97 ± 1.93 ^b^	7.01 ± 2.06 ^b^	18.99 ± 2.21 ^a^	6.52 ± 1.56 ^b^	7.43 ± 1.78 ^b^
Insulin (ng/mL)	1.33 ± 0.29 ^c^	1.82 ± 0.49 ^c^	1.55 ± 0.34 ^c^	3.24 ± 0.59 ^b^	4.54 ± 1.42 ^a^	3.40 ± 0.43 ^b^
Leptin (ng/mL)	0.59 ± 0.38 ^c^	1.61 ± 0.81 ^b^	1.05 ± 0.58 ^b,c^	1.62 ± 0.72 ^b^	2.32 ± 0.90 ^a^	1.40 ± 0.73 ^b^
TG (mg/dL)	56.97 ± 22.37 ^d^	133.51 ± 74.22 ^c^	102.61 ± 63.99 ^c,d^	214.89 ± 78.12 ^b^	273.43 ± 72.97 ^a^	189.71 ± 82.33 ^b^
FFA (mEq/L)	0.81 ± 0.18 ^c^	1.06 ± 0.18 ^b,c^	0.82 ± 0.06 ^c^	1.10 ± 0.20 ^b^	1.39 ± 0.08 ^a^	0.98 ± 0.27 ^b,c^

Values are presented as the mean ± SD. Means with letters (a, b, c, and d) within the column are significantly different from each other, as determined by Duncan‘s multiple range test (*p* < 0.05). SHAM—sham-operated; OVX—ovariectomized; ND—normal diet; HFD—high-fat diet; CA—carnosic acid.
